# Non-Contact Intracardiac Potential Mapping Using Mesh-Based and Meshless Inverse Solvers

**DOI:** 10.3389/fphys.2022.873630

**Published:** 2022-07-07

**Authors:** Shu Meng, Judit Chamorro-Servent, Nicholas Sunderland, Jichao Zhao, Laura R. Bear, Nigel A. Lever, Gregory B. Sands, Ian J. LeGrice, Anne M. Gillis, David M. Budgett, Bruce H. Smaill

**Affiliations:** ^1^ Auckland Bioengineering Institute, University of Auckland, Auckland, New Zealand; ^2^ Department of Mathematics, Universitat Autònoma de Barcelona, Bellaterra, Spain; ^3^ Bristol Heart Institute, University of Bristol, Bristol, United Kingdom; ^4^ HU Liryc, Electrophysiology and Heart Modeling Institute, Fondation Bordeaux Université, Bordeaux, France; ^5^ Centre de Recherche Cardio-Thoracique de Bordeaux, Université Bordeaux, Bordeaux, France; ^6^ INSERM, Centre de Recherche Cardio-Thoracique de Bordeaux, Bordeaux, France; ^7^ Auckland City Hospital, Auckland, New Zealand; ^8^ Department of Medicine, University of Auckland, Auckland, New Zealand; ^9^ Department of Physiology, University of Auckland, Auckland, New Zealand; ^10^ Libin Cardiovascular Research Institute, Calgary University, Calgary, AB, Canada

**Keywords:** atrial fibrillation, open basket catheters, inverse problem, non-contact mapping, endocardial potentials

## Abstract

Atrial fibrillation (AF) is the most common cardiac dysrhythmia and percutaneous catheter ablation is widely used to treat it. Panoramic mapping with multi-electrode catheters has been used to identify ablation targets in persistent AF but is limited by poor contact and inadequate coverage of the left atrial cavity. In this paper, we investigate the accuracy with which atrial endocardial surface potentials can be reconstructed from electrograms recorded with non-contact catheters. An *in-silico* approach was employed in which “ground-truth” surface potentials from experimental contact mapping studies and computer models were compared with inverse potential maps constructed by sampling the corresponding intracardiac field using virtual basket catheters. We demonstrate that it is possible to 1) specify the mixed boundary conditions required for mesh-based formulations of the potential inverse problem fully, and 2) reconstruct accurate inverse potential maps from recordings made with appropriately designed catheters. Accuracy improved when catheter dimensions were increased but was relatively stable when the catheter occupied >30% of atrial cavity volume. Independent of this, the capacity of non-contact catheters to resolve the complex atrial potential fields seen in reentrant atrial arrhythmia depended on the spatial distribution of electrodes on the surface bounding the catheter. Finally, we have shown that reliable inverse potential mapping is possible in near real-time with meshless methods that use the Method of Fundamental Solutions.

## Introduction

Intracardiac catheters can acquire electrograms simultaneously at multiple sites on or close to the heart wall and have been used to construct panoramic maps of electrical activity in patients during persistent atrial fibrillation (AF) (Narayan et al., 2012; Pathik et al., 2018). While macro-scale atrial activation is disorganized in AF, it is argued that repeated patterns of local electrical reentry in such maps may provide targets for the percutaneous catheter ablation procedures used to treat this dysrhythmia (Narayan et al., 2012; Haissaguerre et al., 2016). Effective contact mapping with multi-electrode catheters presents challenges. The spatial distribution of electrodes in the 8-spline basket catheters that have been used for intra-atrial mapping is inherently non-uniform, with greater density along splines than around the equator of these devices when fully deployed (Pathik et al., 2018). Deformation of basket catheter splines in contact with the wall can exacerbate sampling heterogeneity (Pathik et al., 2018). Furthermore, experimental and modelling studies indicate incomplete wall coverage, with ∼50% only of electrodes close to the atrial wall (<5 mm from endocardium) in typical studies of the left atrium (LA) (Oesterlein et al., 2016; Martinez-Mateu et al., 2018; Pathik et al., 2018).

Inverse methods can be used to reconstruct potential maps on the heart surface from electrograms recorded with electrodes that are not in contact with it (Johnson and Bronzino, 2000; Pullan et al., 2005). This requires information about the geometry of the heart surface, the 3D locations of the electrodes and the electrical properties of the volume between them. Mesh-based solutions of the inverse potential problem have been widely used for non-invasive electrocardiographic imaging (ECGi) (Barr et al., 1977; Johnson and Bronzino, 2000; Ramanathan and Rudy, 2001; Pullan et al., 2005; Cluitmans et al., 2017; Duchateau et al., 2019) but also for non-contact intracardiac potential mapping with electrodes arrays mounted on the surface of inflatable balloons (Khoury et al., 1995). To solve this problem, it is necessary to specify Cauchy boundary conditions; that is to assign both potentials and normal potential gradients at points across the boundary on which electrical recordings are made (Johnson and Bronzino, 2000; Pullan et al., 2005). This presents no difficulties for ECGi or for intracardiac inverse potential mapping if electrodes are mounted on an inflatable balloon. Sampling surfaces are insulating in both instances and the normal potential gradient is zero everywhere on them. This is not the case, however, for a multi-electrode basket catheter and normal potential gradients must be estimated on the virtual surface that bounds the electrodes to solve mesh-based formulations of the inverse potential problem. While reliable solutions of the inverse potential problem can in principle be obtained with mesh-based methods such as the finite element method (FEM) or boundary element method (BEM) if appropriate input information is provided (Johnson and Bronzino, 2000; Pullan et al., 2005), meshless methods that employ the Method of Fundamental Solutions (MFS) (Fairweather and Karageorghis, 1998) offer a simpler alternative. The latter approach has been used for ECGi (Wang and Rudy, 2006; Bear et al., 2018) and was recently proposed for non-contact intracardiac potential mapping (Meng et al., 2022).

Here, we provide a systematic *in silico* analysis of mesh-based and meshless methods for solving the intracardiac inverse potential problem—for the first time as far as we are aware. The mathematical bases of the approaches used in this setting are summarized and a simple method for estimating Cauchy boundary conditions from electrograms recorded with a multi-electrode basket catheter is outlined. This is tested in a simplified 2D domain and then used for an FEM-based investigation of inverse potential mapping in the 3D atria. The extent to which accuracy is affected by catheter dimensions, electrode distribution and noise are considered. Finally, we compare the efficacy of this mesh-based approach with meshless methods that use the MFS.

This study demonstrates that reliable non-contact potential mapping can be achieved across a wide range of basket catheter dimensions using mesh-based inverse methods if the electrode distribution is sufficient to provide representative samples of the intracardiac potential field. It also shows that the MFS is equally accurate over most of this range but computationally more efficient.

## Mathematical Background

The electrostatic potential ϕ in a biological volume conductor is typically represented as

$$\nabla \cdot \text{σ}\nabla \phi =\;-I_{v}$$ (1)

where σ is the electrical conductivity tensor and $I_{v}$ is the current per unit volume defined within the solution domain Ω . Electrostatic potentials associated with cardiac electrical activity flow are caused by current flow *via* transmembrane ion channels and transporters in heart muscle cells, but there is no nett current flow elsewhere in the domain. Therefore,

$$\nabla \cdot \text{σ}\nabla \phi =\;0\;\text{in}\;{\text{Ω}}_{\text{H}}$$ (2)

where ${\text{Ω}}_{\text{H}}$ is a heart cavity.

### A Mesh-Based Inverse Approach

**FIGURE 1:**
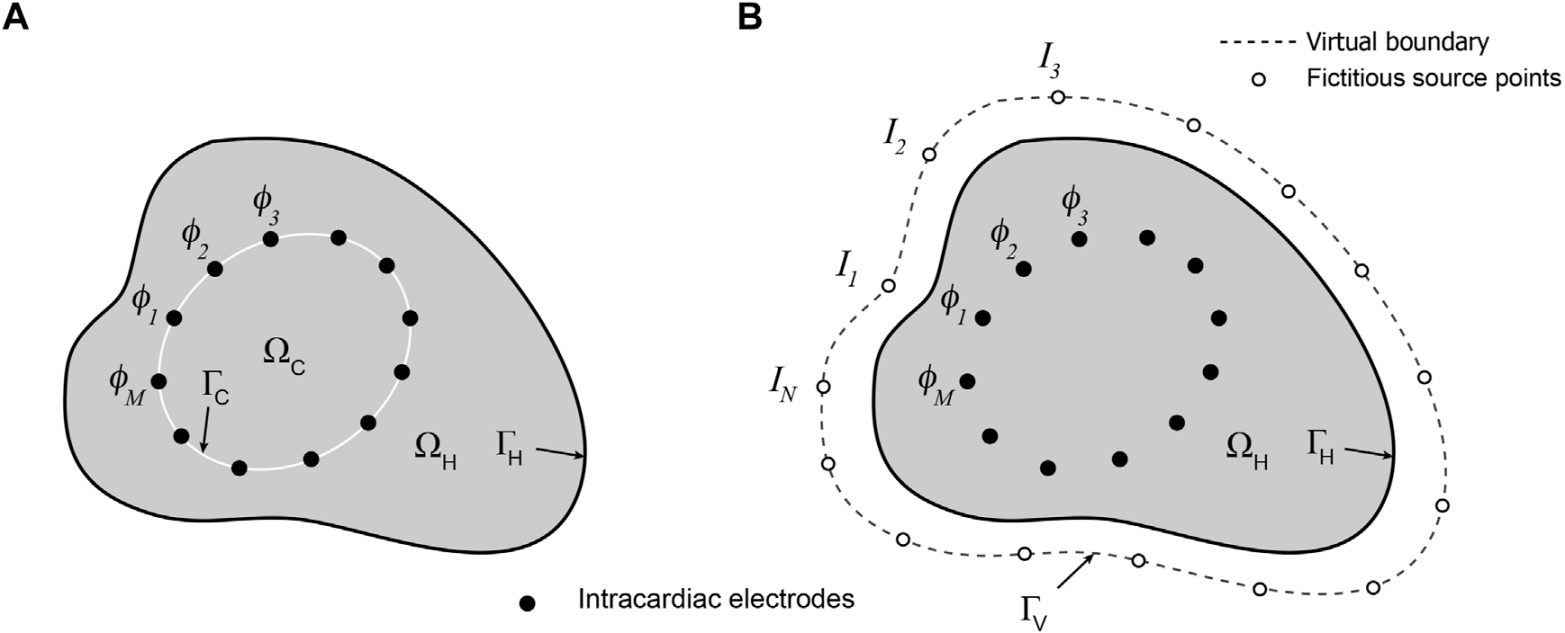
Schematic representations of **(A)** mesh-based and **(B)** meshless/MFS formulations of the intracardiac inverse potential problem which seeks to map the potential distribution on the surface ${\text{Γ}}_{\text{H}}$ that bounds a heart cavity ${\text{Ω}}_{\text{H}}$ from a set of *M* potentials $\phi_{\text{C}}\left(x\right)$ sampled at electrodes inside ${\text{Ω}}_{\text{H}}.$ In **(A)** potentials and normal potential gradients on the surface ${\text{Γ}}_{\text{C}}$ that bounds the electrodes are related to the potential distribution on ${\text{Γ}}_{\text{H}}$ and in solution domain ${\text{Ω}}_{\text{H}}-{\text{Ω}}_{\text{C}}$ . In **(B)** *N* fictitious sources *I*_*i*_ (open circles) distributed around a virtual boundary ${\text{Γ}}_{\text{V}}$ outside ${\text{Γ}}_{\text{H}}$ generate the current flux that gives rise to the potential distribution in ${\text{Ω}}_{\text{H}}.$ These are matched to the sampled potentials $\phi_{\text{C}}\left(x\right)$ enabling potential distribution on ${\text{Γ}}_{\text{H}}$ to be estimated.

A representation of the potential problem is given in Figure 1A. If the potential on the endocardial surface ${\text{Γ}}_{\text{H}}$ is specified (Dirichlet boundary conditions), ϕ can be estimated throughout ${\text{Ω}}_{\text{H}}$ by solving the forward problem Eq. 2.

The objective of the corresponding inverse problem is to reconstruct ϕ on ${\text{Γ}}_{\text{H}}$ from potentials recorded with an array of electrodes introduced into the cavity on a catheter. This can be expressed as a boundary value problem by defining a surface ${\text{Γ}}_{\text{C}}$ that bounds the electrodes on the catheter and encloses the domain ${\text{Ω}}_{\text{C}}$ . We seek to define a set of linear equations that satisfies Eq. 2 in ${\text{Ω}}_{\text{H}}-{\text{Ω}}_{\text{C}}$ and can be reformulated as

$$A\phi_{H}=\phi_{C}$$ (3)

where $\phi_{\text{H}}$ is a vector of data on ${\text{Γ}}_{\text{H}}$ and $\phi_{\text{C}}$ is a vector of data on ${\text{Γ}}_{\text{C}}$ . The inverse problem is to determine $\phi_{\text{H}}$ given $\phi_{\text{C}}$ .

Both problems can be solved numerically using *finite difference*, *finite element* and *finite volume* methods or, because the problem can be reduced to the boundaries alone since σ is uniform and isotropic throughout ${\text{Ω}}_{\text{H}}$ , using *boundary integral* and *boundary element* methods (Oostendorp and van Oosterom, 1991; Johnson and Bronzino, 2000; Pullan et al., 2005). To do so, it is first necessary to discretize the solution domain with an appropriate mesh. Because the inverse problem is ill-posed, solutions are not unique and this amplifies the effects of noise. Tikhonov regularization (Johnson and Bronzino, 2000) is widely used in this setting to reduce instability. It seeks to identify the regularization parameter λ that optimises the objective function

$${\left\|Α\phi_{\text{H}}-\phi_{\text{C}}\right\|}^{2}+\lambda {\left\|L\phi_{\text{H}}\right\|}^{2}$$ (4)

where the first term is the sum of squared residuals from Eq. 3 and the second penalizes lack of smoothness of the solution vector. With zero-order Tikhonov regularization L is the identity matrix (Tikhonov and Arsenin, 1977). The closely related inverse problem of electrocardiography ECGi, in which voltages measured on the torso are used to calculate voltages on the surface of the heart, has been solved using all the numerical methods above (Barr et al., 1977; Johnson and Bronzino, 2000; Pullan et al., 2005; Cluitmans et al., 2017; Bear et al., 2018).

To solve the intracardiac inverse problem, it is necessary to specify appropriate boundary conditions at ${\text{Γ}}_{\text{C}}$ . Continuity of potential and normal current flow is maintained on both sides of the interface (Pullan et al., 2005).

That is

$$\begin{array}{l} \phi_{C}^{in}\;=\;\phi_{C}^{out}\\ {\text{σ}}_{in}\nabla \phi_{C}^{in}\cdot n\;=\;{\text{σ}}_{out}\nabla \phi_{C}^{out}\cdot n \end{array}$$ (5)

where *in* and *out* indicate inner and outer sides of ${\text{Γ}}_{\text{C}}$ respectively.

For a balloon catheter, ${\text{σ}}_{in}=\;\infty$ and $\;\nabla \phi_{\text{C}}\cdot n=0$ , and the inverse problem for this case has been solved using a boundary element method very similar to equivalent approaches used for ECGi (Khoury et al., 1995; Pullan et al., 2005). However, current flows freely across ${\text{Γ}}_{\text{C}}$ with a basket catheter and the dispersion of current in ${\text{Ω}}_{\text{H}}-{\text{Ω}}_{\text{C}}$ can vary substantially between these cases depending on the geometry of ${\text{Γ}}_{\text{H}}$ and ${\text{Γ}}_{\text{C}}$ . The distribution of ϕ in ${\text{Ω}}_{\text{H}}-{\text{Ω}}_{\text{C}}$ reflects this and it follows that ϕ cannot be estimated adjacent to ${\text{Γ}}_{\text{C}}$ unless Cauchy boundary conditions which specify both $\phi_{\text{C}}$ and $\nabla \phi_{\text{C}}.n$ are used. A simple way to set these boundary conditions is to solve the forward problem Eq. 2 for the subdomain ${\text{Ω}}_{\text{C}}$ using $\phi_{\text{C}}$ recorded on ${\text{Γ}}_{\text{C }}$ as Dirichet boundary conditions so that $\phi^{in}$ adjacent to ${\text{Γ}}_{\text{C }}$ can be estimated. Provided that $\phi_{\text{C}}$ samples the potentials on ${\text{Γ}}_{\text{C }}$ adequately, $\nabla \phi_{\text{C}}\cdot n$ can be estimated enabling Cauchy boundary conditions to be specified.

### Meshless Inverse Methods That Use the Method of Fundamental Solutions

The Method of Fundamental Solutions (MFS) provides a means of solving partial differential equations such as the Laplace equation without the need to set up connected internal meshes in the solution domain (Fairweather and Karageorghis, 1998). This approach was applied to ECGi by Wang and Rudy (Wang and Rudy, 2006) and here we extend it to intracardiac inverse potential mapping.

The meshless/MFS formulation of the intracardiac problem is presented in Figure 1B. Potentials ϕ(x)  at points x  in ${\text{Ω}}_{\text{H}}$ are approximated as the linear superposition of source functions positioned at locations ${\left\{\xi_{i}\right\}}_{i=1}^{N}\;$ around a virtual surface ${\text{Γ}}_{\text{V}}$ that encloses ${\text{Ω}}_{\text{H}}$ . It is assumed that the conductivity σ throughout the extended domain bounded by ${\text{Γ}}_{\text{V}}$ is uniform and isotropic, and that the electrical properties of the basket catheter can be neglected.

At any instant, the potential $\phi_{\text{C}}\left(x\right)$ at each of the *M* electrodes at ***x*** in ${\text{Ω}}_{\text{H}}\;$ is estimated as

$$\phi \left(x\right)=\sum_{i=1}^{N}\sigma I_{i}G\left(\xi_{i},x\right)$$ (6)

where $I_{i}=\left(I_{1},\ldots ,I_{N}\right)$ are the source current magnitudes at ${\left\{\xi_{i}\right\}}_{i=1}^{N}$ and *G* is the fundamental solution of the 3D Laplace operator at each point. That is,

$$G\left(\xi ,x\right)=\frac{1}{4\pi \left|\xi -x\right|}$$ (7)

where |ξ−x| is the Euclidean distance between ***x*** and ξ .

This results in an *M* × *N* system of equations and solution of the inverse problem yields the source current magnitudes that best match the $\phi_{\text{C}}\left(x\right)$ recorded with the catheter. The corresponding endocardial potentials $\phi_{\text{H}}\left(x\right)$ can then be reconstructed by evaluating Eq. 6 $\forall \;x\;\in \;{\text{Γ}}_{\text{H}}$ .

This system is inherently under-determined because the number of electrodes *M* is generally less than *N*, the number of fictitious sources needed to map potentials faithfully onto ${\text{Γ}}_{\text{H}}$ .

## Methods

A well-established computational approach (Ramanathan and Rudy, 2001) was used to quantify the accuracy with which potentials around an external boundary can be reconstructed from non-contact potentials sampled within the corresponding domain using inverse solution methods. The basic steps were as follows. First, “ground-truth” potential distributions, one simple and one more complex, were specified on the external boundary. The corresponding internal field was then determined by numerical solution of Laplace’s equation and this potential field was sampled at points corresponding to different catheter dimensions and electrode distributions. Finally, potentials on the outer boundary were reconstructed using the sampled potentials and compared with ground-truth potentials to assess the accuracy of inverse mapping. Key features of our mesh-based inverse approach were tested first with simple 2D problems and then extended to a more realistic 3D FEM analysis using atrial endocardial boundary geometry and representative potential distributions on this anatomy based on experimental measurement and simulation. Finally, the efficacy of inverse potential mapping using a meshless/MFS approach was compared with a representative mesh-based FEM analysis.

### 2D Analysis

**FIGURE 2:**
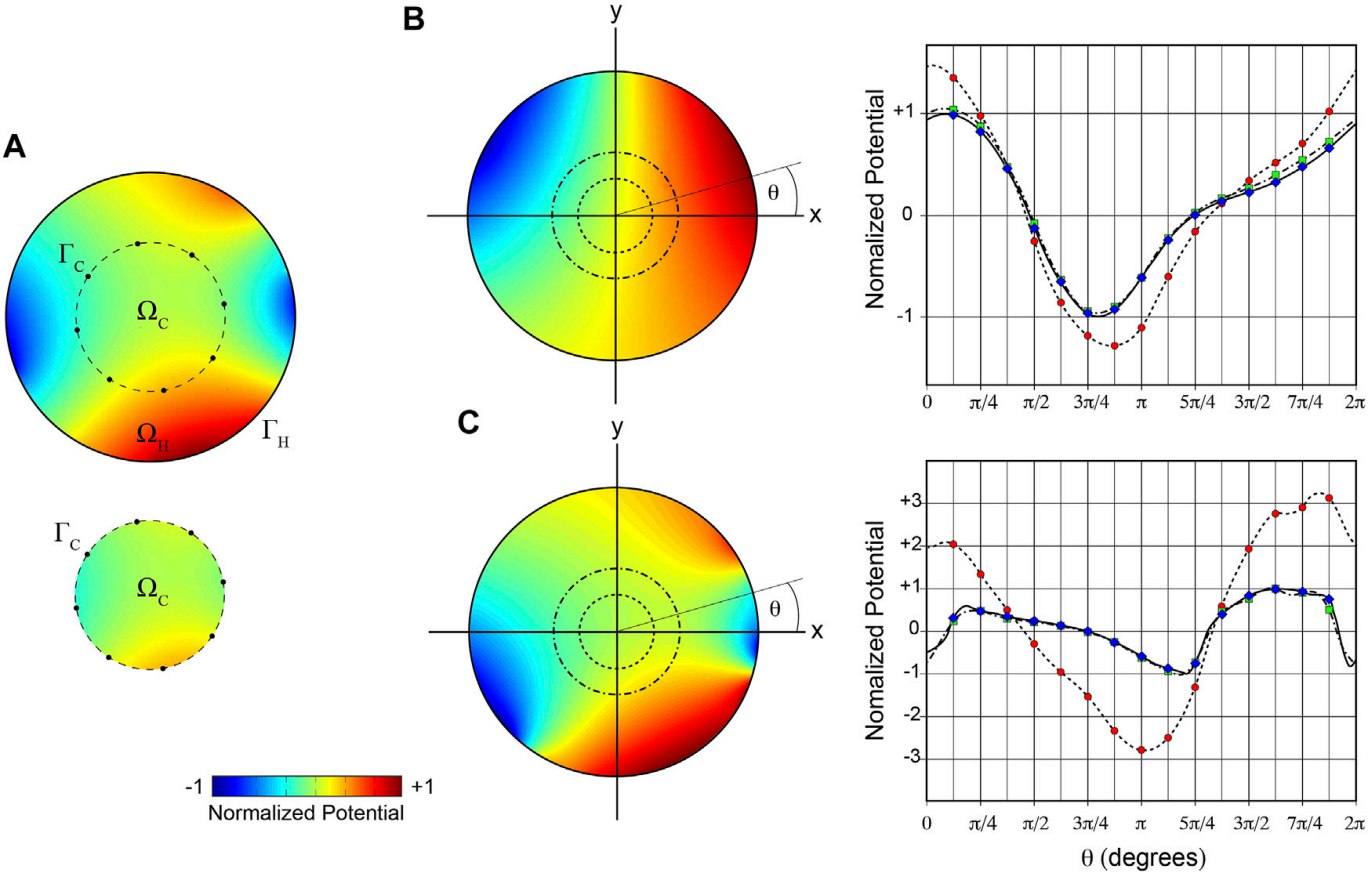
2D illustration of mesh-based inverse potential mapping. **(A)** Schematic of steps in specifying Cauchy boundary conditions on ${\text{Γ}}_{\text{C}}$ . The potential distribution in ${\text{Ω}}_{\text{H}}\;$ (upper panel) is sampled at 8 recording electrodes (black dots). Potentials around ${\text{Γ}}_{\text{C}}$ are reconstructed from these samples with radial basis interpolation and used as Dirichlet boundary conditions in numerical solution of potential distribution in ${\text{Ω}}_{\text{C}}$ (lower panel). This enables estimation of potentials and normal potential gradients around ${\text{Γ}}_{\text{C}}$ . **B** and **(C)** Potential distributions on ${\text{Γ}}_{\text{H}}$ reconstructed from potentials sampled in ${\text{Ω}}_{\text{H}}$ for **(B)** relatively simple, and **(C)** more complex potential fields in ${\text{Ω}}_{\text{H}}$ . Ground-truth potential distributions in ${\text{Ω}}_{\text{H}}$ are given on left and the broken circles indicate the internal boundaries around which samples are acquired (16 sites in both cases). Potentials on ${\text{Γ}}_{\text{H}}$ are reconstructed using Cauchy boundary conditions on ${\text{Γ}}_{\text{C}}$ and compared with ground-truth potentials in the graphs at right. Normalized ground truth surface potentials (solid line, blue diamonds), and surface potentials reconstructed from samples acquired on internal boundaries with relative radii 0.469 (dashed line, green squares) and 0.375 (dotted line, red circles) are plotted as functions of angular coordinate θ.

Aspects of the approach employed here are illustrated in Figure 2. Two different arbitrary ground-truth potential distributions were specified on the boundary ${\text{Γ}}_{\text{H}}$ of the circular domain $\;{\text{Ω}}_{\text{H}}\;$ and the associated potential fields in ${\text{Ω}}_{\text{H}}\;$ were constructed by solving Laplace’s equation with these boundary conditions (Figure 2A) using the finite difference method (FDM) on a polar grid centered on the origin.

Cauchy boundary conditions on ${\text{Γ}}_{\text{C}}$ were determined as shown in Figure 2A. Potentials were sampled at discrete points distributed uniformly on ${\text{Γ}}_{\text{C}}$ which bounds the circular interior domain ${\text{Ω}}_{\text{C}}$ . Potentials around ${\text{Γ}}_{\text{C}}$ were reconstructed using radial-based interpolation and the corresponding potential field in ${\text{Ω}}_{\text{C}}$ was estimated by solving Laplace’s equation with a polar finite difference scheme. Gradients normal to ${\text{Γ}}_{\text{C}}$ were estimated using the FDM with a polar grid centered on the origin of the domains. Transfer Eq. 3 relating $\phi_{\text{H}}$ and $\phi_{\text{C}}$ were formulated using the boundary integral approach developed by Barr et al. (1977) (Barr et al., 1977), then discretized and evaluated as outlined by this group. The inverse problem was solved employing zero-order Tikhonov regularization (Tikhonov and Arsenin, 1977) with the regularization parameter selected using a U-curve algorithm (Chamorro-Servent et al., 2019) based on the discrete Picard condition (Hansen, 2010). This optimizes the singular value decomposition associated with the regularization problem.

### 3D Analyses

Anatomic and experimental data used for 3D analyses were acquired from an anesthetized closed-chest sheep employing methods summarized below. All procedures were approved by the Animal Ethics Committee of the University of Auckland and conform to the Guide for the Care and Use of Laboratory Animals (National Institutes of Health publication no. 85–23).

Gadolinium-enhanced (Gd-DTPA 0.2 mmol kg) ECG-gated magnetic resonance images (MRIs) of the atria (1.0 mm^2^ × 1.0 mm^2^ in-plane resolution approximately parallel to the atrio-ventricular valve plane and 1.6 mm between slices) were acquired with a 3T Siemens Magnetom Skyra scanner in late diastole with lungs inflated. Atrial electrical activation was subsequently mapped using 38 and 48 mm 64-electrode Constellation™ catheters (Boston Scientific) introduced percutaneously into the atria *via* the jugular vein under fluoroscopic guidance. Catheters were positioned in the LA using a guide wire and sheath introduced by trans-septal puncture. Electrograms from LA catheters (bandlimited to 0.5–1,500 Hz and sampled at 3 kHz) were recorded simultaneously in sinus rhythm (SR) using a multi-channel acquisition system (UnEmap, Auckland UniServices) with catheters in different locations. Serial biplane ciné X-ray views of the catheters (LAO/RAO, 25 frames/second, with concurrent Lead II ECG added for synchronization) were acquired immediately after each electrical recording. The ventilator was switched off during fluoroscopy to minimize respiratory motion.

Endocardial surface geometry from a representative LA was segmented from serial MRI using Amira 5.4 (Thermo Fisher Scientific) and reconstructed in 3D with the atrial appendage cropped (see Figure 2). LA electro-anatomic maps were reconstructed for this heart from recordings in SR with 3D electrode locations estimated from biplane X-ray records (Meng et al., 2017). Ground-truth potential distributions in SR were constructed at selected activation times by interpolating potentials around the activation wavefront from recorded electrograms. Ground truth data representing reentrant atrial activation were simulated. Meandering spiral wave reentry was simulated on an isotropic 2D monodomain with Fenton Karma activation kinetics (Fenton and Karma, 1998) using a standard cross-field S1-S2 stimulus protocol (Pandit et al., 2005). Points on the 2D domain were sampled and mapped onto the 3D surface mesh so that surface area was similar in both, with a contour adjacent to the boundary in the former assigned to the mitral valve orifice. Extracellular potentials were approximated from the transmembrane currents computed at each 3D point at a sampling rate of 1 kHz.

The open-source software environment SCIRun (Burton et al., 2011) was used for FEM solutions of 3D forward problems. A triangular surface mesh (1,529 nodes) was fitted to the LA and ${\text{Ω}}_{\text{H}}$ was discretized using tetrahedral elements. Intracardiac potential fields were computed from the ground-truth surface potential distributions by solving Laplace’s equation throughout $\;{\text{Ω}}_{\text{H}}.$ The intracardiac field was sampled at points corresponding to electrodes on two basket catheter configurations with 1) 64 channels with 8 equally spaced electrodes along 8 splines at equal radial angles, and 2) 130 channels with 8 equally spaced electrodes along 16 splines at equal radial angles and electrodes at upper and lower poles. Basket dimensions were uniformly scaled to vary the ratio of catheter volume to LA volume. The centroids of catheters and the LA chamber were aligned to allow maximum catheter expansion and to ensure reproducibility between results. Noise was imposed by adding Gaussian noise independently to the electrograms recorded at each electrode with power set at realistic levels. Signal-to-noise ratio (SNR) is quantified as the ratio of root-mean-squared (RMS) voltages of reconstructed electrograms and noise.

SCIRun was also used for FEM solutions of 3D inverse problems. The methods outlined above for estimating Cauchy boundary conditions for the 2D case were extended to 3D as follows. Intracardiac fields were sampled at points corresponding to electrodes on specified intracardiac catheters. A triangular mesh was fitted to ${\text{Γ}}_{\text{C}}$ (6,720 nodes) and the potential field on this surface was reconstructed from the sampled data using radial-based interpolation. Laplace’s equation was solved in ${\text{Ω}}_{\text{C}}\;$ using these potentials as Dirichlet boundary conditions and ∇ϕ⋅n  was estimated on ${\text{Γ}}_{\text{C}}\;$ with the FDM using a polar grid centered on the catheter. Finally, the volume between boundaries ${\text{Γ}}_{\text{C}}\;$ and ${\text{Γ}}_{\text{H}}\;$ was discretized with a tetrahedral mesh. The inverse problem was solved subject to the potential and normal potential gradient boundary conditions specified on it using zero-order Tikhonov regularization (Tikhonov and Arsenin, 1977) employing the L-curve method to calculate the regularization parameter (Hansen, 2010).

Inverse solutions with the MFS were run with purpose-written code and a more detailed account of the methods used is given in Meng et al. (Meng et al., 2022). In brief, the virtual boundary *Γ*_*v*_ was formed by uniform radial inflation of the atrial surface mesh ${\text{Γ}}_{\text{H}}$ by 6% and individual sources were associated with each of its nodes. Inverse endocardial potential distributions for intracardiac potentials “sampled” with virtual catheters were obtained using zero-order Tikhonov regularization (Tikhonov and Arsenin, 1977) employing the L-curve method to calculate the regularization parameter (Hansen, 2010). Comparisons between FEM and MFS inverse solutions were made at common points on ${\text{Γ}}_{\text{H}}$ .

Correspondence between ground-truth and reconstructed potential maps were quantified by evaluating normalized root-mean-squared error (nRMSE) and correlation coefficient (CC).

$$nRMSE=\;\sqrt{\frac{\sum_{i=1}^{N}{\left(\phi_{GT}^{i}-\;\phi_{R}^{i}\right)}^{2}}{\sum_{i=1}^{N}{\left(\phi_{GT}^{i}\right)}^{2}}}\;\text{and}\;\;CC=\;\frac{\sum_{i=1}^{N}\left(\phi_{GT}^{i}-\;\mu_{GT}\right)\left(\phi_{R}^{i}-\;\mu_{R}\right)}{\sqrt{\sum_{i=1}^{N}{\left(\phi_{GT}^{i}-\;\mu_{GT}\right)}^{2}}\sqrt{\sum_{i=1}^{N}{\left(\phi_{R}^{i}-\;\mu_{R}\right)}^{2}}}$$ (8)

where *N* is the number of surface points compared, $\phi_{GT}^{i}$ and $\phi_{R}^{i}$ are ground-truth and reconstructed potentials at surface point *i*, while $\mu_{GT}\;$ and $\mu_{R}$ are mean values for ground-truth and reconstructed potentials, respectively, across the surface.

Activation times (ATs) for ground-truth and reconstructed electrograms were estimated as maximum negative rate of potential change and the activation time difference ΔT at each surface point was evaluated as the difference between the ground-truth and reconstructed ATs

$$\Delta \mathrm{T=}\left|AT_{\mathrm{GT}}-AT_{R}\right|$$ (9)

SCIRun was used for 3D FEM forward and inverse calculations and for visualization of all 3D results. Meshless/MFS inverse solutions were run in purpose-written C code. All other computation (2D analysis, estimation of potential gradients, regularization and evaluation of correspondence measures), was implemented in the MATLAB programming language (The Mathworks, Natick, Massachusetts).

## Results

### 2D Analysis of Mesh-Based Intracardiac Potential Mapping

We used a simple 2D analysis initially to test the feasibility of our methods for estimating intracardiac Cauchy boundary conditions. Figure 2A illustrates the steps involved. It shows that the ground truth potential field in ${\text{Ω}}_{\text{C}}$ (upper panel) is replicated qualitatively in the lower panel using a limited set of samples around ${\text{Γ}}_{\text{C}}$ . Table 1 presents corresponding median CC and nRMSE for ϕ and ∇ϕ⋅n around ${\text{Γ}}_{\text{C}}$ and demonstrates that both can be estimated with good accuracy in this case. Error increased as ${\text{Γ}}_{\text{C}}\;$ was enlarged relative to ${\text{Γ}}_{\text{H}}$ but was offset by increasing the number of samples.

**TABLE 1:** Effects of number of points on sampling boundary ${\text{Γ}}_{\text{C}}$ represented in Figure 2A and its location relative to outer boundary ${\text{Γ}}_{\text{H}}\;$ on the accuracy with which potentials and normal potential gradients on ${\text{Γ}}_{\text{C}}$ are reconstructed. Potential distribution in ${\text{Ω}}_{\text{H}}$ shown in Figure 2A. ${\text{Γ}}_{\text{C}}$ is concentric with ${\text{Γ}}_{\text{H}}$ and the radius of the former is increased as indicated by the area ratio ${\text{Ω}}_{\text{C}}$ relative to ${\text{Ω}}_{\text{H}}$ . Samples are acquired at 8,16 and 32 uniformly spaced points around ${\text{Γ}}_{\text{C}}$ .

Area ratio		0.049	0.195	0.346	0.541	0.779	0.914	Samples
$\phi \left(x_{j}\right)$	CC	0.9999	0.9995	0.9991	0.9989	0.9984	0.9970	8
	nRMSE	0.0041	0.0101	0.0128	0.0137	0.0170	0.0241	
$\frac{\partial \phi \left(x_{j}\right)}{\partial n}$	CC	0.9996	0.9961	0.9947	0.9977	0.9879	0.9689	
	nRMSE	0.0078	0.022	0.0263	0.0268	0.0280	0.0428	
$\phi \left(x_{j}\right)$	CC	1.0000	1.0000	1.0000	0.9999	0.9997	0.9986	16
	nRMSE	0.0022	0.0020	0.0023	0.0031	0.0078	0.0161	
$\frac{\partial \phi \left(x_{j}\right)}{\partial n}$	CC	0.9998	0.9996	0.9994	0.9989	0.9949	0.9797	
	nRMSE	0.0049	0.0061	0.0079	0.0105	0.0184	0.0347	
$\phi \left(x_{j}\right)$	CC	1.0000	1.0000	1.0000	1.0000	1.0000	0.9999	32
	nRMSE	0.0014	0.0017	0.0020	0.0023	0.0027	0.0046	
$\frac{\partial \phi \left(x_{j}\right)}{\partial n}$	CC	0.9999	0.9997	0.9995	0.9996	0.9996	0.9996	
	nRMSE	0.0030	0.0055	0.0074	0.0062	0.0055	0.0156	

In this figure, we also compare ground-truth potentials on ${\text{Γ}}_{\text{H}}$ with corresponding inverse results reconstructed from samples around internal circles in simple (Figure 2B) and more complex (Figure 2C) fields. Surface potentials reconstructed from samples around an internal radius of 0.469 relative to ${\text{Γ}}_{\text{H}}$ were close to ground-truth (nRMSE 0.02 and 0.06, CC 1.0 and 0.99 for simple and more complex fields, respectively). However, error increased when the dimension of ${\text{Γ}}_{\text{C}}\;$ was reduced further. With a relative radius of 0.375 (∼14% of the domain area), reconstructed surface potentials were overestimated, and the complex surface potential distribution captured less well (nRMSE 0.14 and 0.48, CC 0.99 and 0.70 for simple and more complex fields, respectively). These results demonstrate that mesh-based inverse potential mapping can be used to reconstruct surface potential distributions, but that accuracy is influenced by the dimension of the surface relative to the solution domain.

### 3D Analysis of Mesh-Based Intracardiac Potential Mapping Accuracy

**FIGURE 3:**
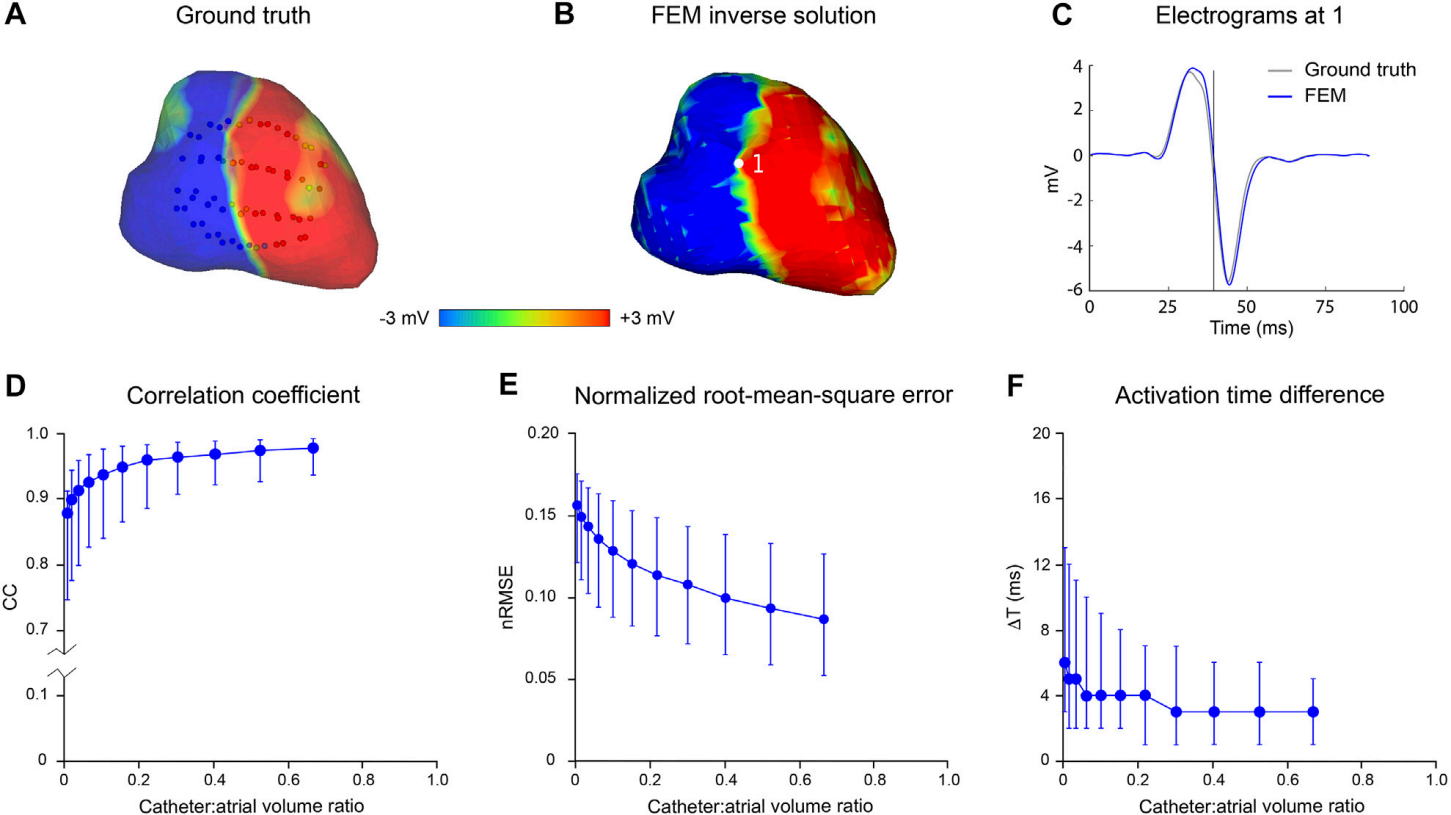
Effect of catheter size on accuracy of inverse potential mapping. Comparison of ground truth potential maps on endocardial surface of LA in SR with inverse maps reconstructed using FEM. Inverse potential maps were reconstructed from electrograms “sampled” using a 64-electrode basket catheter, with centroids of catheter and LA chamber aligned. The upper panel presents typical results for a catheter which bounds a volume fraction of 0.67 relative to LA volume. These include **(A)** ground-truth surface potential distribution 43.9 msec after onset of activation with basket catheter superimposed, and **(B)** corresponding potential maps reconstructed using FEM. Finally, in **(C)** a ground-truth electrogram (black) at point 1 is compared with corresponding electrograms reconstructed using FEM (blue). In the lower panel, **(D)** correlation coefficient (CC) **(E)** normalized root-mean-squared error (nRMSE), and **(F)** activation time difference (ΔT) are presented as functions of relative catheter volume for FEM. Median values and interquartile range are given. Abbreviations: FEM, finite element method; SR sinus rhythm.

Figure 3 presents the results of an *in silico* analysis of the accuracy with which LA surface potential distributions can be reconstructed from non-contact electrograms recorded in SR using 64-channel basket catheters. The ground truth endocardial potential distribution at one instant (43.9 msec after onset of atrial activation) is shown in Figure 3A with the 3D locations of basket catheter electrodes superimposed (the volume ratio of the catheter with respect to LA cavity was 0.67). The corresponding inverse reconstruction of atrial surface potentials in Figure 3B is qualitatively similar to the ground-truth map, while reference and inverse electrograms at a representative site (point 1 in Figure 3B) correspond closely throughout the activation cycle (Figure 3C). Figures 3D–F show acceptable non-contact mapping accuracy for a wide range of catheter dimensions (median: CC >0.96; nRMSE <0.12; ΔT = 3 ms for catheter-atrial volume ratios >0.3). However, error accumulates progressively when catheter dimensions are decreased below this range.

**FIGURE 4:**
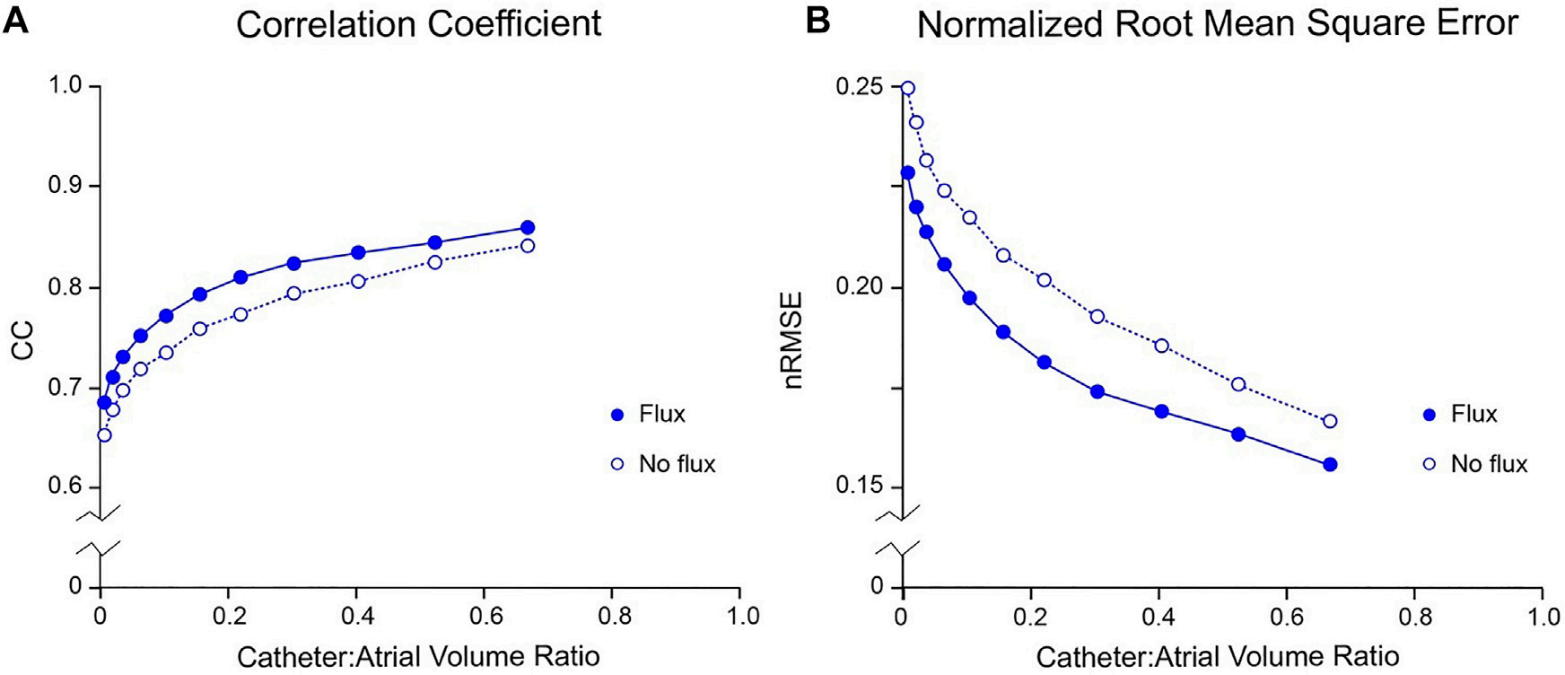
Effect of boundary value specification on accuracy of inverse potential mapping using FEM. Comparison of ground truth potential maps on endocardial surface of LA in SR 43.9 msec after onset of activation with inverse maps reconstructed using FEM from potentials sampled with centrally located internal basket catheters with 64 equi-spaced electrodes. In **(A)** and **(B)**, respectively, relative root-mean-squared error (nRMSE) and correlation coefficient (CC) are presented as functions of catheter volume relative to LA. Additional error introduced by not estimating normal potential gradients on the virtual surface bounding electrodes is indicated by the no flux results (open circles) in which normal potential gradients are set to zero. Abbreviations: FEM, finite element method; SR, sinus rhythm.

Figure 4 presents the error introduced when the normal potential gradient on the surface bounding the electrodes, ${\text{Γ}}_{\text{C}}$ , is not accounted for. In this example, one time-point only is considered (43.9 msec after onset of atrial activation). $\frac{\partial \phi \left(x_{j}\right)}{\partial n}$ is assumed to be zero which corresponds to a no-flux condition at ${\text{Γ}}_{\text{C}}$ . Incorporation of realistic estimates of normal potential gradients on ${\text{Γ}}_{\text{C}}$ reduces nRMSE, with greatest absolute reduction in error for the intermediate range of relative volume ratios. The effects are modest with ∼9% reduction in CC and ∼10% increase in nRMSE at a catheter-atrial volume ratio of 0.3 and absolute error appears to be reduced at the extremes of the relative volume ratio range.

**FIGURE 5:**
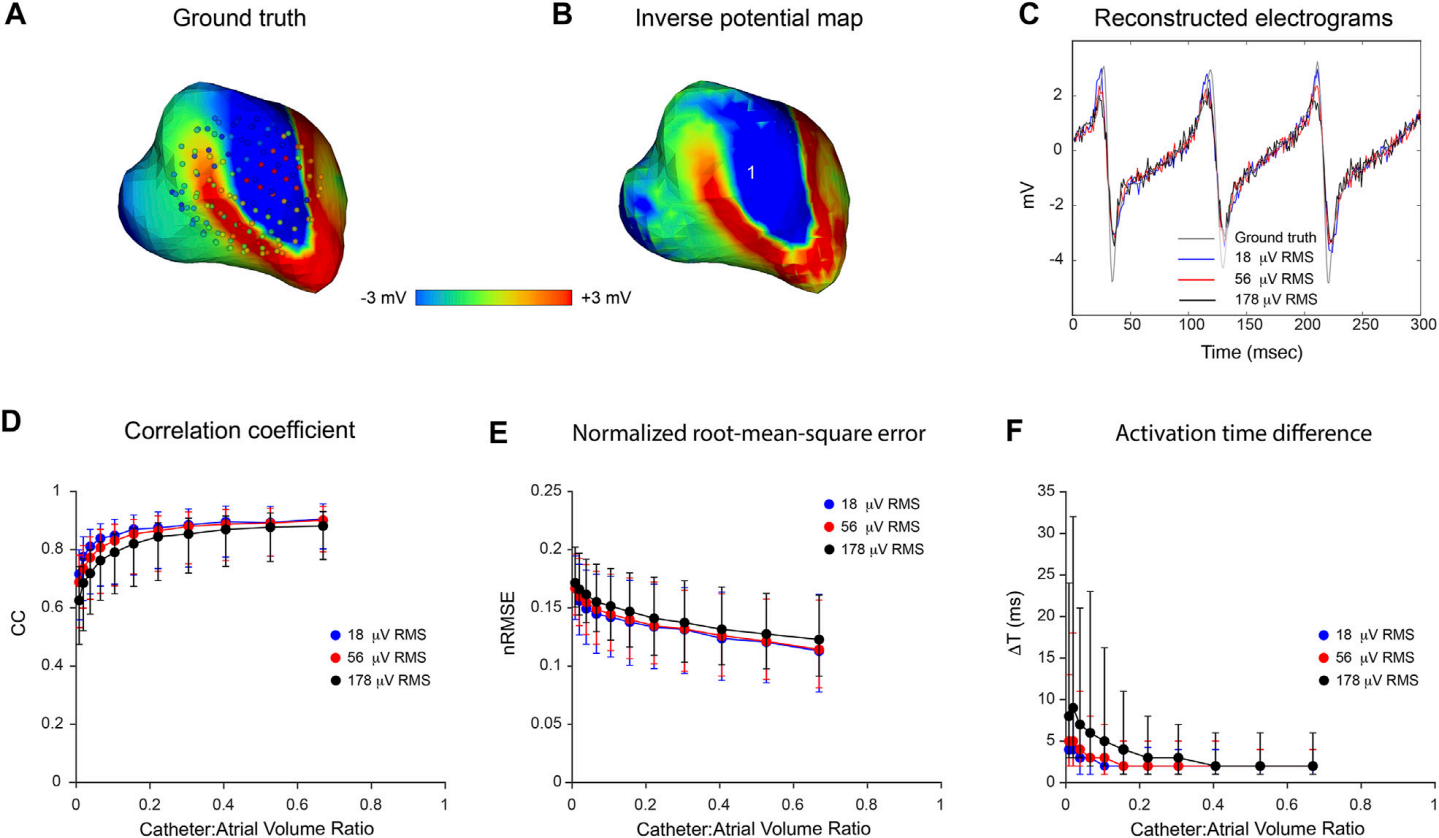
Effects of catheter dimension and noise on inverse potential maps reconstructed during macro-reentry using FEM. LA surface potentials during 3 cycles of simulated atrial flutter are reconstructed from electrograms sampled inside the LA cavity with 130-electrode basket catheters and compared with ground-truth data. The upper panel presents typical results for catheters that bound a volume fraction of 0.67 relative to LA volume. These include **(A)** the ground-truth surface potential distribution at one instant with catheter electrodes overlaid **(B)** corresponding potential map reconstructed using electrograms “sampled” with a 130-electrode basket catheter, and **(C)** electrograms reconstructed at location 1 from sampled records with 18 µV RMS (blue), 56 µV RMS (red) and 178 µV RMS (black) of added Gaussian noise compared with the ground truth electrogram (grey) at the same site. In the lower panel, **(D)** correlation coefficient (CC) **(E)** normalized root-mean-squared error (nRMSE), and **(F)** activation time difference (ΔT) are presented as functions of catheter-atrial volume ratio for these levels of added noise. Median values and interquartile range are given. Abbreviation: FEM, finite element method.

Results of an analysis of inverse mapping accuracy for more complex atrial rhythms in the presence of noise are presented in Figure 5. In this case, a simulated rotor with a moving core was used as ground-truth. Three activation cycles were sampled with a 130-electrode basket catheter and Gaussian noise at RMS voltages of 18, 56 and 178 µV was added to these records. The upper panel shows representative results for a catheter-atrial volume ratio of 0.67. Ground-truth surface potential maps (Figure 5A) were reconstructed with reasonable accuracy in the absence of noise (see Figure 5B). Median results were CC = 0.92, nRMSE = 0.11 and ΔT = 2 ms; clearly better than the corresponding result with a 64-electrode catheter (CC = 0.83, nRMSE = 0.14 and ΔT = 3 ms). At this catheter dimension also, inverse mapping was robust in the presence of realistic levels of electrical noise. Results with systematic variation of relative catheter dimension and noise are shown in Figures 5D–F. Accuracy was relatively invariant despite increasing noise as catheter-atrial volume ratio was reduced from 0.67 to ∼0.2. At dimensions less than this, however, there was a progressive increase in error which scaled with noise level. It is noteworthy that activation time estimates were markedly degraded by noise at reduced catheter dimensions.

An important final observation is that the transfer matrices used for 3D FEM analyses were over-determined in all cases, with the LA represented by a 1529-node triangular surface mesh while a 6720-node triangular mesh was fitted to the catheter. This was necessary to achieve stable solutions.

### Comparison of FEM and MFS Inverse Solutions

**FIGURE 6:**
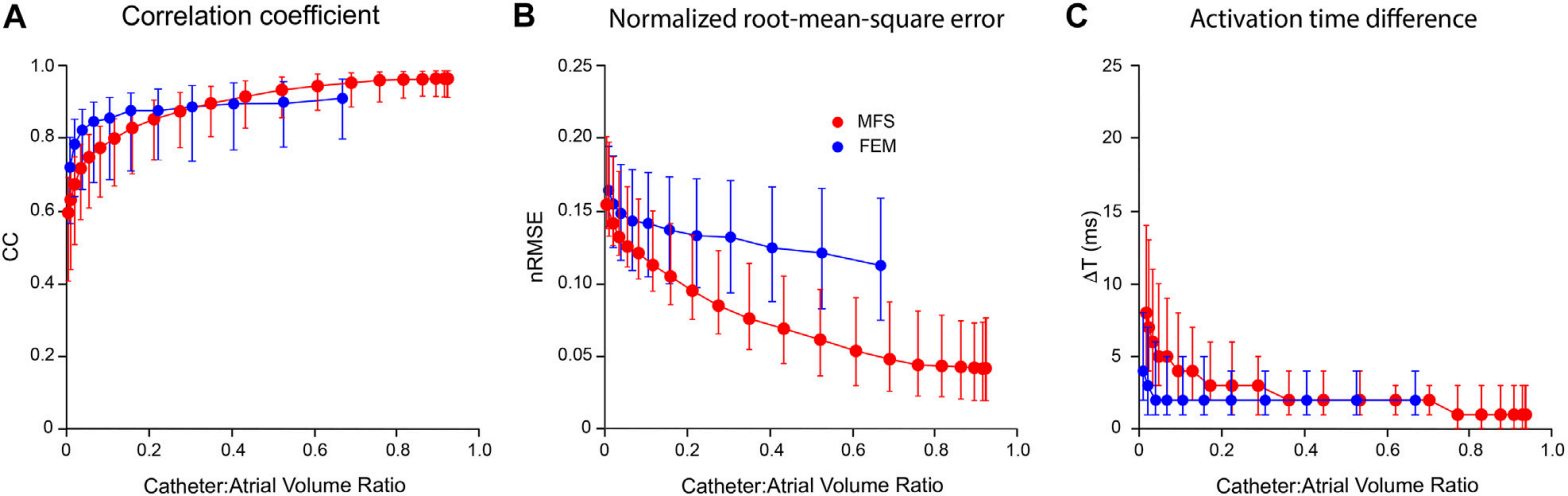
Comparison of inverse potential maps reconstructed during macro-reentry using FEM and meshless methods that employ the MFS. LA surface potentials throughout 3 activation cycles in simulated atrial flutter reconstructed from electrograms sampled inside LA cavity with 130-electrode basket catheters and compared with ground-truth data. **(A)** Correlation coefficient (CC) **(B)** normalized root-mean-squared error (nRMSE), and **(C)** activation time difference (ΔT) are presented as functions of catheter-atrial volume ratio for FEM (blue) and meshless/MFS (red). Median values and interquartile range are given. Abbreviations: FEM, finite element method; MFS method of fundamental solutions.

In Figure 6, we compare the performance of mesh-based inverse mapping employing a FEM solver with a meshless approach that employs the MFS. We used the simulated rotor in Figure 5 as ground-truth and again “sampled” 3 activation cycles with 130-electrode basket catheters of different dimensions. FEM inverse solutions matched ground-truth maps quite well, with median values of CC = 0.91 and nRMSE = 11.3% across the activation sequence at a catheter-atrial volume ratio of 0.67. Corresponding results for the meshless/MFS approach were 0.95 and 4.9%, but activation time differences with ground truth were the same for both. While CC was marginally better with MFS than FEM for catheter-atrial volume ratios >0.3, this measure decreased more rapidly with the MFS when catheter dimensions were reduced further (see Figure 6A). Likewise, ΔT was greater with the MFS for catheter-atrial volume ratios <0.3. In contrast, nRMSE was substantially less for MFS than FEM inverse results across the full volume range.

The main difference between methods was that the MFS was much more efficient computationally than the FEM in our hands. Transfer matrices were simple to set up and inverse solutions were obtained in near real-time using purpose-written code. Finally, the meshless/MFS formulation was robust, with stable inverse solutions despite the fact that transfer matrices were inherently under-determined.

## Discussion

### Summary

In this paper, we present the results of a computational analysis of the accuracy with which endocardial potential maps can be reconstructed from non-contact multi-electrode basket catheter recordings. This inverse problem is addressed initially using a mesh-based approach where transfer relationships are formulated between potentials on the two boundaries involved. This is accurate in principle because assumptions made about the electrical properties of the solution domain are limited (and inherently realistic). However, it requires Cauchy conditions to be specified on the surface ${\text{Γ}}_{\text{C}}$ that bounds the electrodes. A simple and robust way of doing this is outlined and used to solve representative 2D and 3D problems. We demonstrate that effective non-contact intracardiac potential mapping can be achieved using mesh-based methods and that accuracy is determined by 1) the spatial complexity of the intracardiac potential field, 2) the dimensions of the catheter relative to those of the cavity, 3) the distribution of electrodes on the catheter, and 4) the signal-to-noise ratio of the potentials acquired. Finally, we show that a much simpler meshless method which uses the MFS is at least as accurate as mesh-based inverse potential mapping over a wide range of catheter dimensions and computationally far more efficient. This work addresses an important problem in cardiac electrophysiology and is the first *in silico* investigation of this topic, as far as we are aware.

### Mesh-Based Inverse Potential Mapping

With the mesh-based inverse solvers used in this analysis, it is necessary to specify potentials at sufficient points on the surface ${\text{Γ}}_{\text{C}}$ that bounds the electrodes to ensure that the transfer matrices are well-determined. These boundary potentials can be faithfully reconstructed by interpolation if their distribution is represented by the data sampled. This is not sufficient here for complete specification of boundary conditions. It is evident that current flux through an open basket catheter affects the distribution of potentials across the heart cavity and with mesh-based inverse solvers this is captured by specifying normal potential gradients on ${\text{Γ}}_{\text{C}}$ as outlined in the Mathematical Background.

Our 2D analysis demonstrates that intracardiac potential fields in the vicinity of ${\text{Γ}}_{\text{C}}$ can be reconstructed accurately from a relatively small number of potentials sampled uniformly around this boundary. The difference between estimated and expected potentials and normal potential gradients on ${\text{Γ}}_{\text{C}}$ depended on matching the number of electrodes to the spatial complexity of the potential distribution, and correspondence improved as the distance between ${\text{Γ}}_{\text{C}}$ and heart surface ${\text{Γ}}_{\text{H}}$ increased. These findings indicate that it is possible to specify the boundary conditions necessary for non-contact potential mapping using mesh-based inverse solution methods. We have demonstrated that normal potential gradients on ${\text{Γ}}_{\text{C}}$ can be estimated with acceptable accuracy and have shown in Figure 4 that inclusion of this information improves the accuracy of 3D non-contact potential mapping with mesh-based inverse solvers. The robustness of this approach is confirmed by the precision of non-contact potential mapping across a wide range of catheter dimensions in complex rhythms and in the presence of noise (Figures 3, 5).

Our analyses show that the accuracy of inverse potential mapping decreases when catheter dimensions are reduced and this becomes more marked as noise levels are increased. In the 3D examples presented here (Figures 3, 5), error remains relatively low as catheter-atrial volume ratios decrease to ∼0.3 but increases exponentially with further reduction. These findings are intuitively reasonable. With increasing distance from the heart surface, intracardiac potentials are progressively attenuated and smoothed. The extent to which high temporal frequencies on ${\text{Γ}}_{\text{H}}$ can be recovered depends on the regularization method used, but the presence of noise introduces additional problems (Johnson and Bronzino, 2000; Pullan et al., 2005). Because the magnitude of intracardiac electrograms decreases toward the center of ${\text{Γ}}_{\text{H}}$ , the signal-to-noise ratio of records sampled with a small catheter is reduced and the noise is amplified by inverse mapping. Finally, if the catheter is too small it cannot provide an adequate representation of the potentials distributed throughout the cavity, particularly when they are complex spatially.

The 3D analyses above also show that the accuracy with which potentials on ${\text{Γ}}_{\text{H}}$ are reconstructed is improved by matching the number of electrodes to the spatial complexity of the “ground truth” potential distribution. While acceptable non-contact mapping accuracy was achieved in SR using a 64-electrode basket catheter (see Figure 3), a 130-electrode catheter was needed to achieve similar performance for non-stationary reentrant activity (see Figure 5 and related text). If the electrode distribution is not sufficiently dense, high spatial frequencies cannot be recovered and low frequency artefacts (aliasing) may occur (Rice, 1950). This holds for both non-contact and contact mapping.

### Comparison of Mesh-Based and Meshless Inverse Potential Mapping

As noted at the start of the Discussion, we opted to use mesh-based inverse potential mapping as the reference method in this study because assumptions made about the electrical properties of the solution domain with this approach are minimal. We argue that the correspondence of the 3D FEM solutions presented here with ground truth and the stability of these results support this strategy. In contrast, the meshless/MFS alternative with which it is compared employs a much simpler representation of the intracardiac forward problem but introduces additional assumptions about the current sources that give rise to intracardiac potential distributions. The fact that the MFS approach performs better for catheter-atrial volume ratios >0.3 (Figure 6) warrants further consideration. It is likely that much of the apparent improvement with meshless/MFS is due to the compact support for linear interpolation in the FEM implementation used. This gives rise to discontinuities across element boundaries (see Figure 5B) whereas potentials on the heart surface are continuous with meshless inverse mapping. We note that there is no difference in ΔT for catheter-atrial volume ratios >0.3 and argue that meshless/MFS inverse potential mapping is at least as accurate as mesh-based inverse methods over this range.

The major advantage of meshless/MFS methods in this setting is that the forward transfer function is computationally simple and can be assembled very rapidly. In contrast, with mesh-based alternatives, such as FEM, the forward transfer function is complex and time consuming to assemble and invert. Furthermore, our results indicate that the meshless/MFS representation of the intracardiac problem is much better conditioned and therefore more robust than FEM. This is reflected by the fact that an over-determined transfer matrix was needed for stable inverse solutions with FEM, whereas accurate solutions were obtained with MFS despite the fact that transfer matrices were under-determined.

### Potential Clinical Impact of These Findings

Non-contact intracardiac mapping systems that have been used clinically have utilized balloon-mounted multi-electrode array for potential mapping (Khoury et al., 1995; Khoury et al., 1998; Schilling et al., 1999) or have reconstructed membrane charge density from electrograms recorded with an open basket catheter (Willems et al., 2019). While the inverse problem techniques used are different, one would expect the information recovered to be affected similarly by electrode density and positioning, and catheter size, i.e. the number of recording electrodes, their physical spacing on the catheter and proximity of the electrodes to the atrial wall once the catheter is fully deployed. Validation studies have shown that the accuracy with which endocardial electrograms are constructed with the first of these approaches is inversely related to the distance from the electrodes array to corresponding points on the cavity surface (Earley et al., 2006). As far as we are aware, an equivalent systematic validation has not been completed for the second. This study indicates that reliable non-contact potential mapping can also be performed using multi-electrode catheters and could be carried out in near real-time using meshless methods that employ the MFS.

In terms of optimal catheter design, greater electrode density and more uniform distribution would be expected to provide higher resolution. However, the question of how much is enough has only started to be addressed recently. Martinez et al. (Martinez-Mateu et al., 2018) showed computationally that methods used to transform basket electrogram signals back into catheter surface potential maps may result in the creation of fictitious repetitive activation patterns resembling AF rotors when the input information was too sparsely sampled. Williams et al. (Williams et al., 2018) on the other hand defined optimal endocardial sampling densities, both computationally and *in-vivo*, required to resolve activation patterns of varying complexities. They report that a minimum endocardial sampling density of 1.0–1.5 points/cm^2^ is required, with higher densities needed to resolve spiral wave activity. Whilst they were looking at endocardial interpolation of contact recordings not inverse solutions, it is evident from our work here that potential pitfalls in inverse mapping also need to be addressed with good catheter design and mechanistic insight.

### Limitations

It could be argued that the BEM is better matched to the mesh-based inverse potential problem addressed here (Oostendorp and van Oosterom, 1991; Johnson and Bronzino, 2000; Pullan et al., 2005). The FEM generates sparse transfer matrices and is computationally expensive, while BEMs reduce the solution domain to the boundaries only giving rise to compact transfer matrices that can reduce computational overheads and improve accuracy (Johnson and Bronzino, 2000; Pullan et al., 2005). However, our purpose here was to benchmark the mesh-based approach and we opted to use FEM to avoid possible instability that can occur when boundaries are geometrically complex as is the case in the atria. We note that our mesh-based analysis has proved stable and that the meshless/MFS methods with which they are compared are much more efficient computationally than either FEM or BEM. A further limitation is that although our ground-truth data represent atrial rhythms of increasing complexity they do not replicate the spatio-temporal disorder that characterizes AF.

## Conclusion

This computational analysis indicates that potentials on the endocardial surface of a cardiac chamber can be reconstructed with intracardiac multi-electrode basket catheters using inverse solution methods provided that the boundary geometry is specified and the 3D location of catheters with respect to it are known. These data are now available clinically. Panoramic electro-anatomic maps can therefore be generated at successive time steps from non-contact recordings. Mapping accuracy is determined by 1) the distance of recording electrodes from the endocardium, 2) their distribution within the subdomain sampled, and 3) rhythm complexity. These issues should be factored into the design of future non-contact multi-electrode basket catheters. We conclude that reliable non-contact potential mapping can be carried out in near real-time using meshless methods that employ the MFS.

## Data Availability

The original contributions presented in the study are included in the article/Supplementary Material, further inquiries can be directed to the corresponding author.
